# Comparison of Lower Airway Sampling Strategies in Children with Protracted Bacterial Bronchitis (CLASSIC-PBB): a non-randomised, internally controlled, multicentre trial

**DOI:** 10.1136/thorax-2025-224292

**Published:** 2026-03-18

**Authors:** Francis J Gilchrist, Ivonne Solis-Trapala, Mathew Aspey, Zoe Baker, Robert Bowler, Malcolm Brodlie, Seema Desai, Caroline Harris, Emily Hinton, Hemant Kulkarni, Simon Lea, Aviva Ogbolosingha, Ian Sinha, Joanne Stock, Will Carroll, CLASSIC PBB study group, Alice Alcock

**Affiliations:** 1 Department of Paediatric Respiratory Medicine, University Hospitals of North Midlands NHS Trust, Stoke-on-Trent, UK; 2 School of Medicine, Keele University, Newcastle-under-Lyme, UK; 3 Research and Innovation Directorate, University Hospitals of North Midlands NHS Trust, Stoke-on-Trent, UK; 4 Department of Paediatric Respiratory Medicine, Great North Children’s Hospital, Newcastle upon Tyne, UK; 5 Translational and Clinical Research Institute, Faculty of Medical Sciences, Newcastle University, Newcastle upon Tyne, England, UK; 6 Department of Microbiology, University Hospitals of North Midlands NHS Trust, Stoke-on-Trent, UK; 7 On Behalf of Patient and Public Involvement and Engagement for CLASSIC PBB, University Hospitals of North Midlands NHS Trust, Stoke-on-Trent, England, UK; 8 Department of Paediatric Respiratory Medicine, Sheffield Children’s Hospital NHS Foundation Trust, Sheffield, England, UK; 9 Research and Development, University Hospitals of North Midlands NHS Trust, Stoke-on-Trent, Staffordshire, UK; 10 Department of Respiratory Medicine, Alder Hey Children’s NHS Foundation Trust, Liverpool, England, UK; 11 Academic Department of Paediatrics, University Hospitals of North Midlands NHS Trust, Stoke-on-Trent, England, UK; 12 University Hospitals of North Midlands NHS Trust, Stoke-on-Trent, England, UK; 13 Newcastle Upon Tyne Hospitals NHS Foundation Trust, Newcastle upon Tyne, England, UK; 14 Sheffield Children’s Hospital, Sheffield, England, UK; 15 Alder Hey Children’s NHS Foundation Trust, Liverpool, England, UK

**Keywords:** Bronchoscopy, Paediatric Lung Disease, Bacterial Infection

## Abstract

**Background:**

Protracted bacterial bronchitis (PBB) is a leading cause of paediatric chronic wet cough. Identifying the causative pathogen promotes antibiotic stewardship, but bronchoalveolar lavage taken during flexible bronchoscopy (FB-BAL) is the only validated sampling strategy. We prospectively investigated if cough swab (CS) or induced sputum (IS) was a viable alternative.

**Methods:**

Children with PBB, referred for FB-BAL, provided a cough swab and induced sputum. Discordance in microbiological yield (failure to identify identical pathogens) between cough swab and BAL, and between induced sputum and BAL was assessed. This was performed for single-lobe (BAL1), two-lobe (BAL2) and six-lobe BAL (BAL6).

**Results:**

135 underwent FB-BAL, 134 produced a cough swab and 111 an induced sputum sample. 30 (22%) cough swab, 67 (60%) induced sputum and 101 (78%) BAL6 were pathogen positive. cough swab/BAL6 discordance was seen in 74% with a discordance OR (95% CI) for cough swab^−^BAL6^+^ versus cough swab^+^BAL6^−^ of 30.3 (9.6 to 95.8). induced sputum/BAL6 discordance was seen in 71% with a discordance OR (95% CI) for induced sputum^−^BAL6^+^ versus induced sputum^+^BAL6^−^ of 4.2 (2.4 to 7.4). The ability of each sampling strategy to identify all pathogens from cough swab, induced sputum and BAL6 was: cough swab 17%, induced sputum 38%, BAL1 40%, BAL2 53% and BAL6 78%. The false negative rates were: cough swab 67%, induced sputum 27%, BAL1 33%, BAL2 16% and BAL6 9%.

**Conclusions:**

The pathogen yield of both cough swab and induced sputum had high discordance with BAL6, meaning neither is a viable alternative to FB-BAL. However, the tolerability and relatively low false negative rate of induced sputum mean it can be part of early PBB assessment in children who do not warrant FB-BAL.

**WHAT IS ALREADY KNOWN ON THIS TOPIC**

- Protracted bacterial bronchitis (PBB) is an endobronchial infection and the leading cause of chronic wet cough in children. Identifying the pathogen promotes antibiotic stewardship, but the only validated lower airway sampling strategy in PBB is bronchoalveolar lavage taken during bronchoscopy. This is reserved for severe cases, meaning most antibiotic prescribing is done ‘blind’ without microbiological data.

**WHAT THIS STUDY ADDS**

- The microbiology yield and false negative rate of induced sputum (IS) samples were similar to that of single-lobe BAL. As induced sputum can be easily performed in the outpatient clinic, it has a role in the early investigation of children with suspected PBB, who do not warrant bronchoscopy. Cough swabs performed poorly and have no role.

**HOW THIS STUDY MIGHT AFFECT RESEARCH, PRACTICE OR POLICY**

- Widespread use of induced sputum as an early investigation in PBB has the potential to support more targeted antibiotic prescribing, including narrower-spectrum therapy in selected cases. It would also potentially reduce the need for flexible bronchoscopy, which is burdensome for the child and their family.

## Introduction

Protracted bacterial bronchitis (PBB) is a chronic endobronchial bacterial infection and a common cause of persistent wet cough in young children.^1^ It is most prevalent in those aged 1–5 years and is the leading cause for referrals to paediatric respiratory clinics in developed countries.^2 3^ The cough affects all aspects of the child’s life, including sleep, educational performance and physical activity.^4^ This has a detrimental effect on the child’s quality of life and that of family members.^5^ The diagnosis of PBB is based on: (1) continuous wet cough >4 weeks’ duration; (2) absence of ‘specific cough pointers’ suggestive of another diagnosis; and (3) cough resolution following 2 weeks of an appropriate oral antibiotic.^6^ By definition, cough resolution in PBB occurs with antibiotic treatment^3 7^ but relapses are common.^8–10^ Those with recurrent PBB (>3 episodes in 12 months) are at increased risk of future bronchiectasis.^11–13^ This is associated with life-long morbidity and increased healthcare utilisation.^14^

Although not currently part of the diagnostic criteria, identifying the causative pathogen in PBB allows a narrower spectrum antibiotic to be prescribed in around 40% of cases, decreasing the risk of antimicrobial resistance (AMR).^8 13^ Commonly identified organisms include: *Haemophilus influenzae*, *Streptococcus pneumoniae*, *Moraxella catarrhalis* and *Staphylococcus aureus*.^8 15^ Sampling the lower airway in PBB is not straightforward. Affected children rarely expectorate sputum and the only validated sampling strategy is bronchoalveolar lavage taken during flexible bronchoscopy (FB-BAL).^8 15^ This is invasive, requires a general anaesthetic and causes significant disruption to families.^16^ Due to the large number of children presenting with suspected PBB, it is impractical to perform FB-BAL in all cases. In most centres, FB-BAL is reserved for those whose cough does not resolve with antibiotics or who have recurrent PBB.^17^ The majority of antibiotic prescribing in PBB is therefore undertaken without microbiology data, promoting the widespread use of broad-spectrum antibiotics.^18^

If a less invasive strategy could successfully sample the lower airways of children with PBB, it could be performed in the outpatient clinic providing microbiology data for more children and earlier in their patient journey. This would reduce the need for FB-BAL enabling antibiotic prescribing to be targeted, reducing AMR. Although PBB trials have not suggested a role for upper airway cultures (nasopharyngeal and/or oropharyngeal swabs),^19–21^ data from trials in children with cystic fibrosis (CF) suggest there may be a role for induced sputum (IS) samples.^22^ The potential role for induced sputum in PBB is supported by a study showing comparable culture results between BAL and bronchial aspirates.^23^ Comparison of Lower Airway Sampling Strategies in Children (CLASSIC) PBB examined pathogen yield discordance between cough swab (CS), induced sputum and single-lobe (BAL1), two-lobe (BAL2) and six-lobe BAL (BAL6) to clarify how non-invasive methods capture pathogens and to identify differences in sensitivity.

## Methods

### Study design

CLASSIC PBB was a non-randomised, internally controlled, multicentre trial in which the pathogen yield from three lower airway sampling strategies was compared in children with PBB. The protocol was published during the recruitment phase.^24^

### Participants

Children aged 1–10 years with a diagnosis of PBB, referred for a clinically indicated FB-BAL were prospectively recruited from four UK tertiary paediatric respiratory centres. These were: Alder Hey Children’s Hospital (Liverpool), Great North Children’s Hospital (Newcastle upon Tyne), Sheffield Children’s Hospital and Staffordshire Children’s Hospital at Royal Stoke (Stoke on Trent). Children were excluded if they had bronchiectasis, CF or immunodeficiency, if the FB-BAL was being performed for a therapeutic indication (ie, lobar collapse) or if the parent/guardian was a non-English speaker and translation facilities were insufficient to guarantee informed consent. Potential participants were identified in the outpatient clinic by the delegated local team. The study was discussed with the family and, if interested, they were provided with the participant and parent information sheets. Written informed consent was obtained in clinic or on the ward, prior to FB-BAL. When appropriate, informed assent was obtained from the child.

### Procedures

The cough swab and induced sputum were obtained by a delegated and appropriately trained physiotherapist or clinician, on the same day but prior to the FB-BAL. FB-BAL was not a study procedure but performed using a standardised, BAL6 methodology.^25^ The methodologies for cough swab, induced sputum and FB-BAL are detailed in the protocol.^24^ The total volume of lavage fluid used during FB-BAL ranged from 3 to 6 mL/kg. The cough swab was always taken before the induced sputum to avoid any impact on the microbiology yield of the cough swab from the hypertonic saline. When CLASSIC PBB opened to recruitment, restrictions related to aerosol generating procedures were in place due to the COVID-19 pandemic. Local NHS Trust guidelines regarding the screening of children prior to admission and the use of personal protective equipment during the procedures varied throughout the study and between centres but were strictly adhered to. All samples were cultured by the local UK Accreditation Service certified microbiology laboratory and the results made available to the local team.

### Outcomes

#### Primary outcome

The primary outcome was discordance in pathogen yield between cough swab and BAL6, and between induced sputum and BAL6. The pathogen yield was deemed discordant if exactly the same organism(s) were not identified in both samples. Discordance between cough swab and BAL6 was classified as cough swab^+^/BAL6^−^ when pathogens were isolated on cough swab but not BAL6 and cough swab^−^/BAL6^+^ when pathogens were isolated on BAL6 but not cough swab. Discordance between induced sputum and BAL6 was defined in the same way.

#### Secondary outcomes

1. The sensitivity of cough swab and induced sputum to identify all pathogens isolated on BAL6.
2. The sensitivity of cough swab and induced sputum to identify each of the most common four pathogens isolated on BAL6.
3. Concordance/discordance in pathogen yield between paired cough swab/BAL and induced sputum/BAL samples when BAL data limited to:
  - BAL1 in which only pathogens isolated from the right middle lobe (RML) or most affected lobe were included in the analysis.
  - BAL2 in which only pathogens isolated from RML/most affected lobe and left lingula lobe were included.
4. The ability of cough swab, induced sputum, BAL1, BAL2 and BAL6 to individually identify the combined pathogen yield from cough swab, induced sputum and BAL6.
5. Success/failure of induced sputum to produce a usable sample.
6. Tolerability of the three sampling strategies.

### Procedural burden

A four-question, procedural burden survey using five-point Likert scales was completed by the parent/guardian (with input from the child when appropriate) after each of the three procedures. The questions related to preprocedure worry, discomfort during the procedure, postprocedure side effects and overall tolerability.

### Statistical analysis

The sample size was calculated using McNemar’s test^21^ for paired proportions, reflecting the primary outcome of discordance between paired samples. Assuming a proportion of discordant pairs of 0.37 (based on CF-Sputum Induction Trial (CF-SPIT)^22^), we required a sample size of 110 to detect a ratio of discordant proportions (identification of an organism on BAL6 not isolated on induced sputum versus identification of an organism(s) on induced sputum not identified on BAL6) of 3, with 90% power and 0.05 significance. Assuming an induced sputum success rate of 84%,^22^ we needed a sample size of 131. The sample size required to detect a difference between cough swab and BAL6 was expected to be smaller due to expected higher discordance.

For analysis, conditional logistic regression was used to account for the paired structure and to estimate ORs with 95% CIs, providing interpretable measures of association between paired observations. We developed conditional logistic regression models to estimate the OR for discordant pathogen detection between cough swab and BAL6, and between induced sputum and BAL6. Discordance was defined as either: (1) one or more pathogens identified on BAL6 but not on cough swab (or induced sputum), or (2) one or more pathogens identified on cough swab (or induced sputum) but not on BAL6. Similar conditional logistic regression models were developed to assess the discordance in pathogen detection for each of the four the most common pathogens (*Haemophilus influenzae, Staphylococcus aureus, Streptococcus pneumoniae and Moraxella catarrhalis*). Additionally, the sensitivity and two-sided 95% score CIs were estimated for each paired test. Bangdiwala agreement charts illustrating levels of concordance and sensitivity were presented.^26^

The concordance and discordance between the pathogen yield of cough swab and BAL1, BAL2 and BAL6, as well as induced sputum and BAL1, BAL2 and BAL6, were summarised descriptively. Similarly, the ability of cough swab, induced sputum, BAL1, BAL2 and BAL6 to identify the combined pathogen yield from cough swab, IS and BAL6 was summarised using frequency tables.

Two logistic regression models were used to describe the association of age, sex and centre with four indicators of induced sputum success (ability to tolerate, successfully obtain a sample, requirement for suction and pathogen positivity). The first model explored the association of age with the probability of the outcome, and the second model adjusted for sex and centre. For this analysis, age was treated as a categorical variable determined by tertile thresholds (1–2, 2–4 and >4 years) enhancing the interpretability of results which were qualitatively equivalent to including age as a continuous variable. The Likert score for tolerability was described using a frequency table stratified by age group.

The statistical analysis was performed using the statistical software R.

## Results

### Recruitment

Between February 2022 and March 2024, 137 children were recruited. Slight delays in confirming recruitment of participants to the study organisers by some centres meant we recruited past the planned sample size of 131. The indication for the FB-BAL was recurrent or quickly relapsing PBB. All children had not had any antibiotics for at least 14 days. Their baseline characteristics are shown in table 1.

**Table 1:** Baseline characteristics and sample collection

Recruited children	137
Females	69 (50.3%)
Males	68 (49.7%)
Age (median (IQR))	3.0 (2.0–4.5) years
Duration of cough (median (IQR))	13 (10–24) months
Previous antibiotics (median (IQR))	4 (3–7) courses

### Sample collection

The parents of one child withdrew consent, and one had their FB-BAL cancelled after providing a cough swab and induced sputum. Of the 135 FB-BALs, 130 had all six lobes sampled, 4 only had results for BAL2 and 1 for BAL1. A cough swab was successfully obtained from 134 (99%) children (two did not tolerate). induced sputum was attempted in 131 (5 declined) with 111 (85%) successfully producing a sample. Eight (6%) did not tolerate the induced sputum and another 12 (9%) tolerated the procedure but did not produce sputum. Suction was used in 95 (86%) of the children who produced an induced sputum sample. The number of paired samples was: cough swab/BAL1 133, cough swab/BAL2 132, cough swab/BAL6 127, induced sputum/BAL1 110, induced sputum/BAL2 109, induced sputum/BAL6 105. Triplicate samples (cough swab, induced sputum and BAL6) were obtained in 105 children. See figure 1.

**Figure 1:**
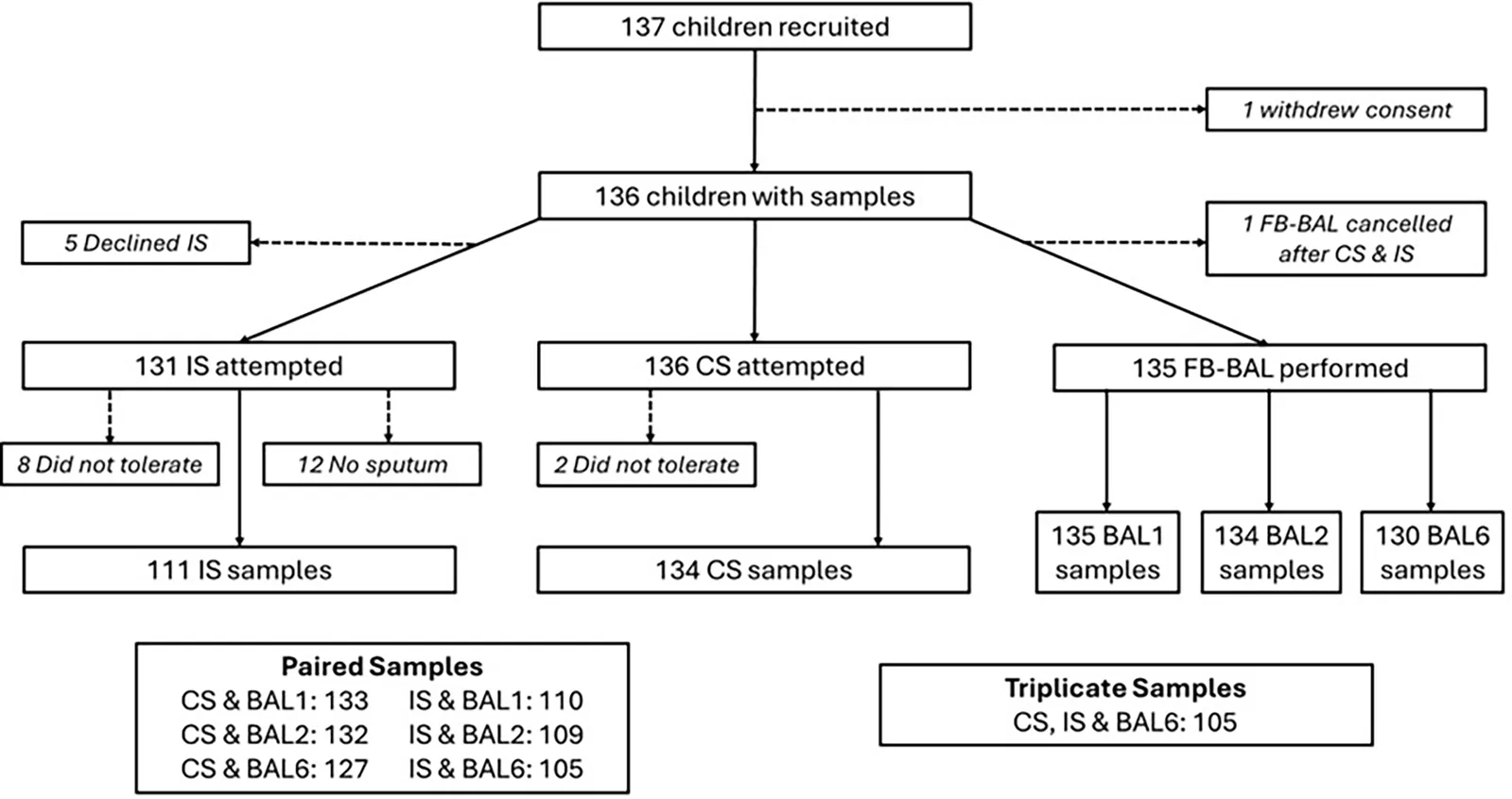
Participant flow chart. BAL, bronchoalveolar lavage; BAL1, single-lobe BAL; BAL2, two-lobe BAL; BAL6, six-lobe BAL; CS, cough swab; FB-BAL, bronchoalveolar lavage taken during flexible bronchoscopy; IS, induced sputum.

### Microbiology

The following organisms were excluded from analysis as they are not PBB pathogens: *Acinetobacter baumannii, Acinetobacter haemolyticus, Aeromonas punctata, Aspergillus species, Chryseobaterium indologenes, Haemophilus haemolyticus and Pseudomonas putida*. The excluded organisms were only isolated once or twice and never from BAL samples. 28 (21%) cough swab and 67 (60%) induced sputum samples were pathogen positive, isolating 30 and 89 pathogens, respectively (table 2). There was no difference in the per cent of pathogen positive samples for induced sputum obtained with and without the need for suction (58/95 (61%) vs 9/16 (56%), p=0.716). The pathogens isolated from the different sampling strategies are shown in online supplemental table 1. The most common isolates were *Haemophilus influenzae, Moraxella catarrhalis, Streptococcus pneumoniae* and *Staphylococcus aureus*.

**Table 2:** Summary of samples

	CS	IS	BAL1	BAL2	BAL6
Obtained samples	134	111	135	134	129
Pathogen positive	28 (20.9%)	67 (60.4%)	77 (57.0%)	98 (73.1%)	101 (78.3%)
No. of pathogens identified	30	89	96	151	182
Mixed flora	57 (42.5%)	18 (16.2%)	24 (17.8%)	37 (27.6%)	52 (40.3%)

Mixed flora: multiple bacteria identified (often from the upper respiratory tract) without a predominant species.

BAL1, single-lobe BAL; BAL2, two-lobe BAL; BAL6, six-lobe BAL; BAL, bronchoalveolar lavage; CS, cough swab; IS, induced sputum.

### Pathogen yield discordance and sensitivity of cough swab and induced sputum to identify BAL6 pathogens

The pathogen yields of cough swab and BAL6 were discordant in 94/127 (74%) of paired samples with a discordance OR for CS^−^BAL6^+^ versus cough swab^+^BAL6^−^ of 30.3 (95% CI 9.6 to 95.8). The sensitivity of cough swab to detect all pathogens on BAL6 was 8%. The pathogen yields of induced sputum and BAL6 were discordant in 75/105 (71%) of paired samples with a discordance OR for induced sputum^−^BAL6^+^ versus induced sputum^+^BAL6^−^ of 4.0 (95% CI 2.3 to 7.0) based on 105 paired samples. The sensitivity of induced sputum to detect all pathogens on BAL6 was 22%. The primary outcome for induced sputum was recalculated using BAL2/BAL1 data as reference for five samples in which BAL6 was not available, thereby ensuring the minimum sample size of 110 was met. This gave a discordance OR of 4.2 (95% CI 2.4 to 7.4) and a sensitivity of 23%. See table 3. The associated agreement plots are shown. See figure 2. The sensitivities of induced sputum to detect *Staphylococcus aureus, Haemophilus influenzae, Moraxella catarrhalis* and *Streptococcus pneumoniae* were 54%, 50%, 40% and 19%. The corresponding sensitivities for cough swab were 23%, 12%, 3% and 4%. These data, together with discordance, discordance OR and concordance, are shown in table 3 and their agreement plots are shown in online supplemental figure 1.

**Table 3:** Discordance, discordance OR and concordance between the evaluation test (IS or CS) and BAL6, together with sensitivity of the evaluation test for all and each of the four the most common pathogens

Sampling strategy	Pathogen	Discordance		Discordance OR (95% CI)	Concordance		Sensitivity (95% CI)
		IS^−^BAL6^+^	IS^+^BAL6^−^		IS^+^BAL6^+^	IS^−^BAL6^−^	
**IS**	*All pathogens (n=105*)	60	15	4 (2.3 to 7.0)	17	13	22% (14% to 33%)
	*All pathogens (n=110)* †	63	15	4.2 (2.4 to 7.4)	19	13	23% (15% to 34%)
	*H. influenzae*	29	9	3.2 (1.5 to 6.7)	29	38	50% (37% to 63%)
	*S. pneumoniae*	21	5	4.5 (1.7 to 12.2)	5	74	19% (7% to 40%)
	*S. aureus*	6	1	2.8 (0.8 to 10.7)	7	91	54% (26% to 80%)
	*M. catarrhalis*	21	6	3.5 (1.4 to 8.7)	14	64	40% (24% to 58%)
		**CS** ^**−**^ **BAL6** ^**+**^	**CS** ^**+**^ **BAL6** ^**−**^		**CS** ^**+**^ **BAL6** ^**+**^	**CS**^**−**^ **BAL6**^**−**^	
**CS**	*All pathogens*	91	3	30.3 (9.6 to 95.8)	8	25	8% (4% to 16%)
	*H. influenzae*	51	1	27.1 (9.5 to 77.1)	7	46	12% (5% to 24%)
	*S. pneumoniae*	25	0	NA*	1	79	4% (0% to 22%)
	*S. aureus*	10	3	4.5 (1.1 to 18.7)	3	89	23% (6% to 54%)
	*M. catarrhalis*	34	0	NA*	1	70	3% (0% to 17%)

The discordance OR compares discordant pairs where BAL6 is positive (BAL6+) and the evaluation test is negative (IS− or CS−) to pairs where BAL6 is negative and the evaluation test is positive (IS+ or CS+). This was estimated using conditional logistic regression.

* The OR estimate was not available in two cases where there were no discordant BAL6−IS+ pairs.

† Data from BAL2/BAL1 was used for five paired samples when BAL6 was not available (ensuring minimum sample size of 110 pairs was met).

BAL, bronchoalveolar lavage; BAL1, single-lobe BAL; BAL2, two-lobe BAL; BAL6, six-lobe BAL; CS, cough swab; *H. influenzae*, *Haemophilus influenzae*; IS, induced sputum; *M. catarrhalis*, *Moraxella catarrhalis*; *S. aureus*, *Staphylococcus aureus*; *S. pneumoniae*, *Streptococcus pneumoniae*.

**Figure 2:**
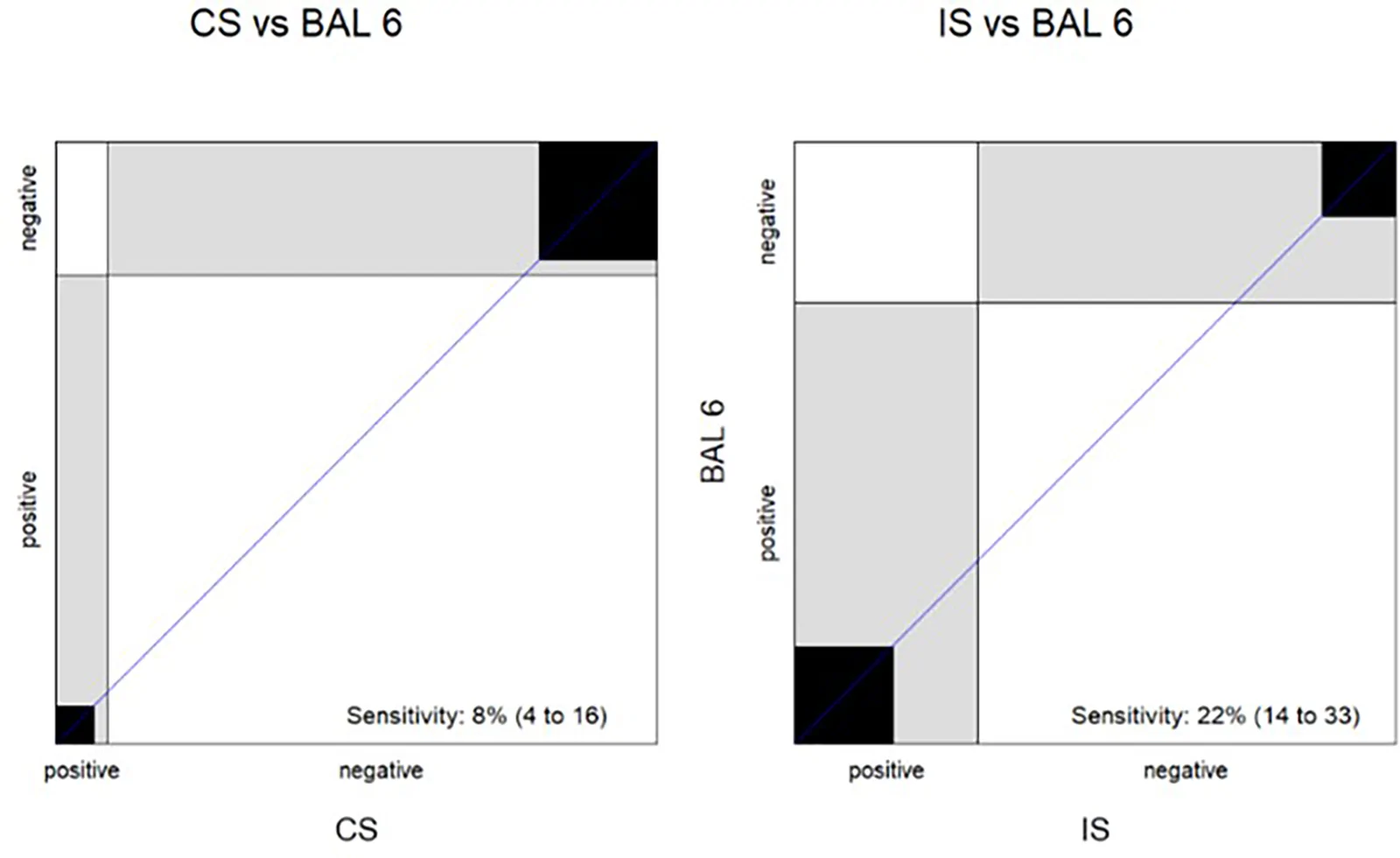
Agreement charts comparing two methods (IS or cough swab) against the reference standard BAL6 in detecting one or more pathogens, along with test sensitivity (95% CI). Each chart visualises concordance (black boxes) within the maximum possible agreement (grey rectangles). The bottom-left corner black box highlights true positive concordance; here, the height of the black box relative to the grey rectangle height reflects the sensitivity of the test. Discordant results are represented by black boxes falling short of grey areas across the chart. BAL 2 was used as a reference method for five IS versus BAL comparisons where BAL 6 was not available. BAL, bronchoalveolar lavage; BAL2, two-lobe BAL; BAL6, six-lobe BAL; cough swab, cough swab; IS, induced sputum.

### Ability to detect all pathogens

A total of 178 pathogens were isolated from the 105 triplicate samples (cough swab, induced sputum and BAL6). 31 pathogens not isolated on BAL6 were found on either cough swab or induced sputum (22 IS, 7 cough swab and 2 both). See figure 3. The ability of cough swab and induced sputum to identify all pathogens isolated on cough swab, induced sputum and BAL6 was 17% and 38%, respectively. This compared with 40%, 53% and 78% for BAL1, BAL2 and BAL6. See table 4. The false negative rates for each sampling strategy (no pathogens isolated from that strategy despite other samples being positive) are also shown in table 4.

**Table 4:** Data on 105 triplicate cough swab, IS and FB-BAL6 samples

	CS	IS	BAL1	BAL2	BAL6	All combined
Number (%) pathogens identified	24 (13.5)	81 (45.5)	81 (45.5)	111 (62.4)	147 (82.6)	178 (100)
Identified all pathogens from cough swab, IS and BAL6	18 (17.1%)	40 (38.1%)	42 (40.0%)	56 (53.3%)	82 (78.1%)	105 (100%)
False negatives	70 (66.7%)	28 (26.7%)	35 (33.3%)	17 (16.2%)	9 (8.6%)	0

False negatives: (no pathogen isolated from that sampling strategy despite pathogen(s) being identified by other samples strategies).

BAL, bronchoalveolar lavage; BAL1, single-lobe BAL; BAL2, two-lobe BAL; BAL6, six-lobe BAL; CS, cough swab; FB-BAL, bronchoalveolar lavage taken during flexible bronchoscopy; IS, induced sputum.

**Figure 3:**
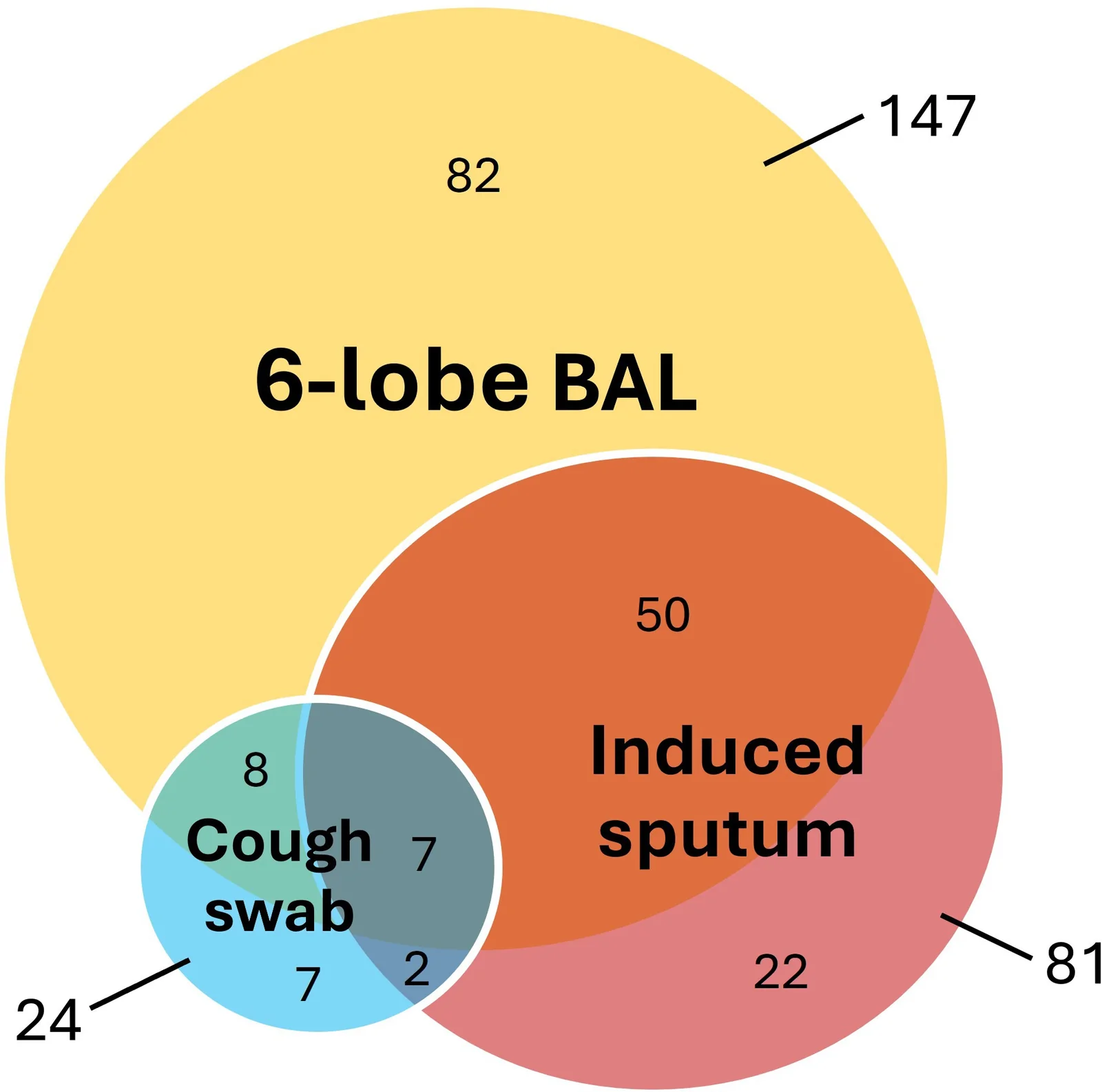
Venn diagram showing which sampling strategy identified the 178 pathogens from 105 triplicate samples. BAL, bronchoalveolar lavage.

### Factors influencing the success of induced sputum

When compared with children aged 1–2 years, the odds of requiring suction were significantly lower for children aged 2–4 years (OR=0.1 (0.01 to 0.8), p=0.034) and children aged >4 years (OR=0.08 (0.01 to 0.7), p=0.021). There was weak evidence for lower odds of pathogen positivity in children aged >4 years compared with those aged 1–2 years (OR=0.4 (0.2 to 1.1) p= 0.087) and the association was no longer apparent after adjusting for sex and centre. Age did not affect the odds of tolerating induced sputum or of successfully obtaining a sample.

### Parental reported burden associated with the sampling strategies

Parents reported the most pre-procedure burden from FB-BAL with 39% worrying ‘often’ or ‘nearly always’ compared with 7% from cough swab and 8% from induced sputum. FB-BAL also had the highest postprocedure burden with 9% reporting ‘moderate’ or ‘severe’ side effects compared with 1% with cough swab and 0% with induced sputum. Discomfort during the procedures was highest for induced sputum, with 26% reporting this as ‘moderate’ or ‘severe’ compared with 8% for cough swab and 11% for FB-BAL. 21% reported the induced sputum as ‘difficult to tolerate’ or ‘intolerable’ compared with 10% for cough swab and 5% for FB-BAL. A table showing the full breakdown of the parental reported burden scores is shown in online supplemental table 2.

## Discussion

CLASSIC PBB was undertaken to investigate the potential application of alternative lower airway sampling strategies in children with PBB that are less burdensome and less invasive than FB-BAL. The use of such strategies has the potential to increase the number of children with PBB in whom microbiology data is available, promoting antibiotic stewardship. To the authors’ knowledge, this is the first study designed to systematically characterise the microbiology yield and tolerability of different lower airway sampling strategies in children with PBB. The organisms cultured are in keeping with previously published data.^15 27^ Both cough swab and induced sputum had high levels of discordance with BAL6 and low sensitivities. Neither can therefore be used as a viable alternative to FB-BAL in children with PBB. The extremely low pathogen yield of cough swab and its high false negative rate mean that as well as not being a viable alternative to FB-BAL, it also has no clear role in the early assessment of children with PBB. In contrast, induced sputum had a higher pathogen yield and a relatively low false negative rate, performing similarly to BAL1. Most children were able to provide an induced sputum sample and it was generally well tolerated. It can therefore easily be performed in clinic, suggesting induced sputum can be a useful investigation in the early assessment of children with PBB who do not warrant FB-BAL.

Both cough swab and induced sputum had high levels of discordance with BAL6, but the cause of this differed between the two sampling strategies. For cough swab, the vast majority of the discordance was caused by the cough swab being falsely negative as reflected by its high discordance OR. This is in keeping with a previous study which compared the microbiology yield of BAL and nasopharyngeal swabs.^27^ The discordance OR for induced sputum was lower as a larger proportion of the discordance was due to induced sputum identifying pathogens not found on BAL6. The percentage of pathogen positive induced sputum samples was almost three times higher than that for cough swab and slightly higher than BAL1. While it did not perform as well as BAL2 and BAL6, this demonstrates its potential role in PBB. The incremental increase in the number of pathogen positive procedures and the total number of pathogens from BAL1 to BAL2 and BAL6 highlights the heterogeneity of pathogen distribution in PBB and suggests BAL6 should be standard practice when trying to identify the causative pathogen. This supports previously published data in both CF^22 28^ and PBB.^15^ The identified pathogens were in keeping with previously studied pathogens,^15^ with *Haemophilus influenzae, Moraxella catarrhalis, Streptococcus pneumoniae and Staphylococcus aureus* accounting for 57% of cough swab isolates, 93% of induced sputum isolates and 89% of BAL6 isolates. Interestingly, there were marked differences in the sensitivity of cough swab and induced sputum to identify the different pathogens. Most notably was the low sensitivity (19%) of *Streptococcus pneumoniae* culture from induced sputum samples, compared with 40–54% for the three other the most common pathogens.

Although BAL6 is usually assumed to be the gold standard when sampling the lower airway, 22 pathogens were identified on induced sputum that were not isolated on the paired BAL6 samples. This suggests that in some cases, IS samples can identify a causative PBB pathogen missed on FB-BAL. This phenomenon was also seen in the CF-SPIT study which compared the microbiology yield from different sampling strategies in children with CF.^22^ Interestingly, some of the organisms missed on BAL6 in CF-SPIT were the most pathogenic organisms including *Pseudomonas aeruginosa* and *Burkholderia cepacia* complex. CLASSIC PBB isolated *Pseudomonas aeruginosa* in four children. This was identified on BAL in all four children (one did not have BAL6) and on cough swab and induced sputum in three. Due to CF-SPIT identifying pathogens on induced sputum missed by BAL6, they reported the sensitivity of IS and BAL6 using a gold standard of all pathogens isolated from these two sampling strategies. These were calculated as 63% and 81%, respectively.^22^ Using the same methodology (by excluding cough swab data), the sensitivity of induced sputum and BAL6 in CLASSIC PBB was 47% and 86%. The reason induced sputum did not perform as well in CLASSIC PBB as it did in CF SPIT needs more investigation but is likely to reflect the different disease processes in PBB and CF, as well as those in our study being younger (3.0 vs 8.2 years).

Induced sputum has not previously been used in children with PBB. This is possibly due to concern that their young age makes it less likely they will successfully produce a usable sample. However, in this cohort, the success rate of induced sputum at producing a mucoid sample was 85%, with 6% not tolerating the procedure and 9% failing to produce a sample. This is similar to the success rate in CF^22^ (84% in 124 children, median age 8.2 years) and tuberculosis^29^ (95% in 149 children, median age 9 months). Although only 6% of children were unable to complete the induced sputum, one in five parents reported the procedure as difficult to tolerate or intolerable. It is therefore important to counsel parents and children carefully before an induced sputum is attempted. Parents reported less preprocedure worry and fewer postprocedure side effects from induced sputum and cough swab than with FB-BAL.

The authors acknowledge there are a number of limitations to this study. FB-BAL is not a standard investigation for PBB. The children in this study were referred for a clinically indicated FB-BAL due to recurrent or quickly relapsing PBB and therefore represent the more severely affected end of the PBB spectrum. CLASSIC PBB was focused on discordance and was not powered to demonstrate equivalence or clinical interchangeability of induced sputum with the gold standard; accordingly, findings should be interpreted with caution. A concordance-focused study design would represent an important complementary approach. We also did not collect prospective data on which antibiotics were prescribed post-FB-BAL.

CLASSIC PBB has shown cough swab and induced sputum samples can be successfully obtained from children with PBB. The low sensitivity of both cough swab and induced sputum and their high discordance with the pathogen yield from BAL6 samples means they are not viable alternatives to FB-BAL. Despite this, the relatively low false negative rate and the ease with which it can be performed in the outpatient clinic suggests induced sputum can have a role in the early investigation of children with suspected PBB who do not warrant FB-BAL. Access to microbiological data from induced sputum has the potential to support more targeted antibiotic prescribing, including narrower-spectrum therapy in selected cases. The use of IS in this way may reduce the number of FB-BALs performed on children with PBB, which would reduce the burden on families and reduce healthcare costs. While induced sputum is much cheaper than FB-BAL, it still requires time, expertise and resources which have cost implications. FB-BAL continues to have a role in those with recurrent PBB or a negative induced sputum.

## Supplementary material

10.1136/thorax-2025-224292 online supplemental file 1

10.1136/thorax-2025-224292 online supplemental file 2

## Data Availability

Data are available upon reasonable request.
