# Supplementary material for: Comparison of Lower Airway Sampling Strategies in Children with Protracted Bacterial Bronchitis (CLASSIC-PBB): a non-randomised, internally controlled, multicentre trial
Source: Thorax. 2026 Mar 18;81(11):e224292. doi: 10.1136/thorax-2025-224292 (PMC13691722; doi:10.1136/thorax-2025-224292)
Supplement: Supplementary file 1 [file thorax-81-11-s001.pdf]

Supplementary Table 1: Prevalence of isolated pathogens from the different sampling strategies.

	**CS** (n=134)	**IS** (n=111)	**BAL1** (n=135)	**BAL2** (n=134)	**BAL6** (n=130)
Haemophilus influenzae	9	42	34	64	70
Moraxella catarrhalis	1	21	24	36	43
Streptococcus pneumoniae	1	11	17	24	32
Staphylococcus aureus	6	9	10	12	17
Pseudomonas aeruginosa	3	3	4	4	3
Enterobacter cloacae	3	0	0	0	4
Serratia liquefaciens	1	0	2	2	2
Beta Haemolytic *Streptococcus*	0	0	2	2	2
Klebsiella oxytoca	1	0	1	1	2
Streptococcus dysgalactiae	1	1	1	1	1
Escherichia Coli	0	0	0	2	2
Serratia marcescens	2	1	0	0	0
Streptococcus pyogens	0	0	1	1	1
Group G Streptococcus	0	1	0	0	1
Methicillin resistant *Staphylococcus aureus*	0	0	0	1	1
Haemophilus parainfluenzae	0	0	0	1	1
Klebsiella pneumonia	1	0	0	0	0
Group A *Streptococcus*	1	0	0	0	0

Data refer to the number of recruits with each pathogen. Due to some samples not being obtained in all children, the numbers of samples are different for each sampling strategy.

Supplementary Figure 1: Agreement plots for the four commonest pathogens showing concordance and discordance between the evaluation test (IS or CS) and the paired BAL6 samples, along with test sensitivity.

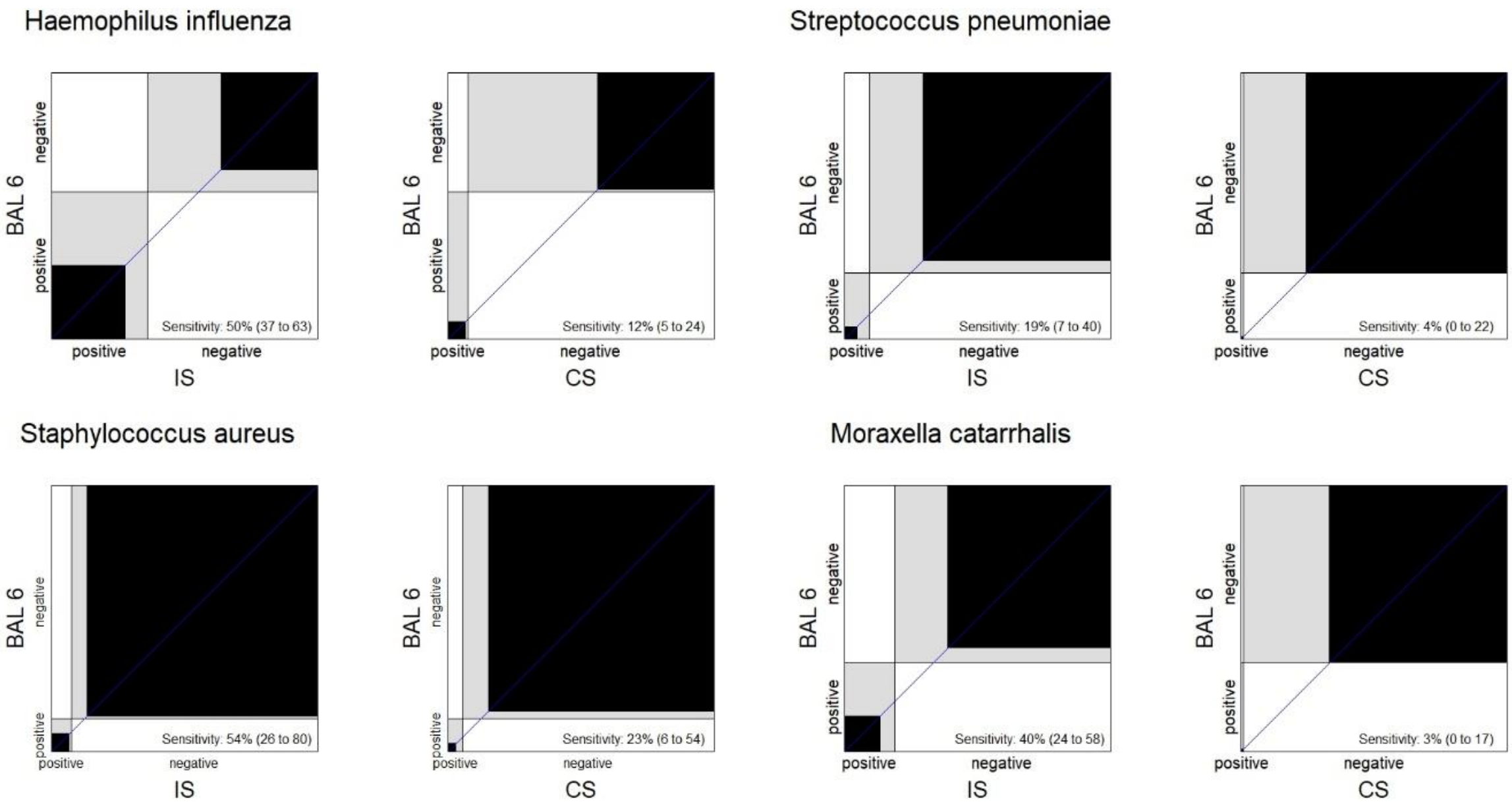

Supplementary Table 2: Parental reported burden associated with the sampling strategies:

		CS	IS	BAL
**Pre-procedure worry?**	Not at all	72 (53·7%)	58 (45·3%)	14 (10·6%)
	Rarely	34 (25·4%)	35 (27·3%)	23 (17·4)
	Occasionally	19 (14·2%)	25 (19·5%)	44 (33·3%)
	Often	5 (3·7%)	6 (4·7%)	36 (27·3%)
	Nearly always	4 (3·0%)	4 (3·1%)	15 (11·4%)
**Discomfort during the procedure?**	None	60 (44·8%)	30 (23·3%)	52 (42·6%)
	Very mild	38 (28·4%)	27 (20·9%)	35 (28·7%)
	Mild	26 (19·4%)	39 (30·2%)	22 (18·0%)
	Moderate	8 (6·0%)	29 (22·5%)	13 (10·7%)
	Severe	2 (1·5%)	4 (3·1%)	0
**Post-procedure side effects?**	None	120 (89·6%)	97 (75·8%)	40 (30·8%)
	Very mild	11 (8·2%)	24 (18·8%)	47 (36·2%)
	Mild	2 (1·5%)	7 (5·5%)	31 (23·8%)
	Moderate	1 (0·7%)	0	11 (8·5%)
	Severe	0	0	1 (0·8%)
**Overall tolerability**	Very easy	49 (36·6%)	19 (14·6%)	17 (13·1%)
	Easy	45 (33·6%)	33 (25·4%)	49 (37·7%)
	Bearable	26 (19·4%)	51 (42·3%)	57 (43·8%)
	Difficult	11 (8·2%)	18 (13·8%)	7 (5·4%)
	Intolerable	3 (2·2%)	9 (6·9%)	0
