# Supplementary material for: Comparison of Lower Airway Sampling Strategies in Children with Protracted Bacterial Bronchitis (CLASSIC-PBB): a non-randomised, internally controlled, multicentre trial
Source: Thorax. 2026 Mar 18;81(11):e224292. doi: 10.1136/thorax-2025-224292 (PMC13691722; doi:10.1136/thorax-2025-224292)

Comparison of Lower Airway Sampling Strategies in Children with Protracted Bacterial Bronchitis (CLASSIC-PBB): A non-randomised, internally controlled, multicentre trial

Gilchrist FJ et al. Thorax-2026. DOI: 10.1136/thorax-2025-224292

Venn diagram showing which sampling strategy identified the 178 pathogens from 105 triplicate samples

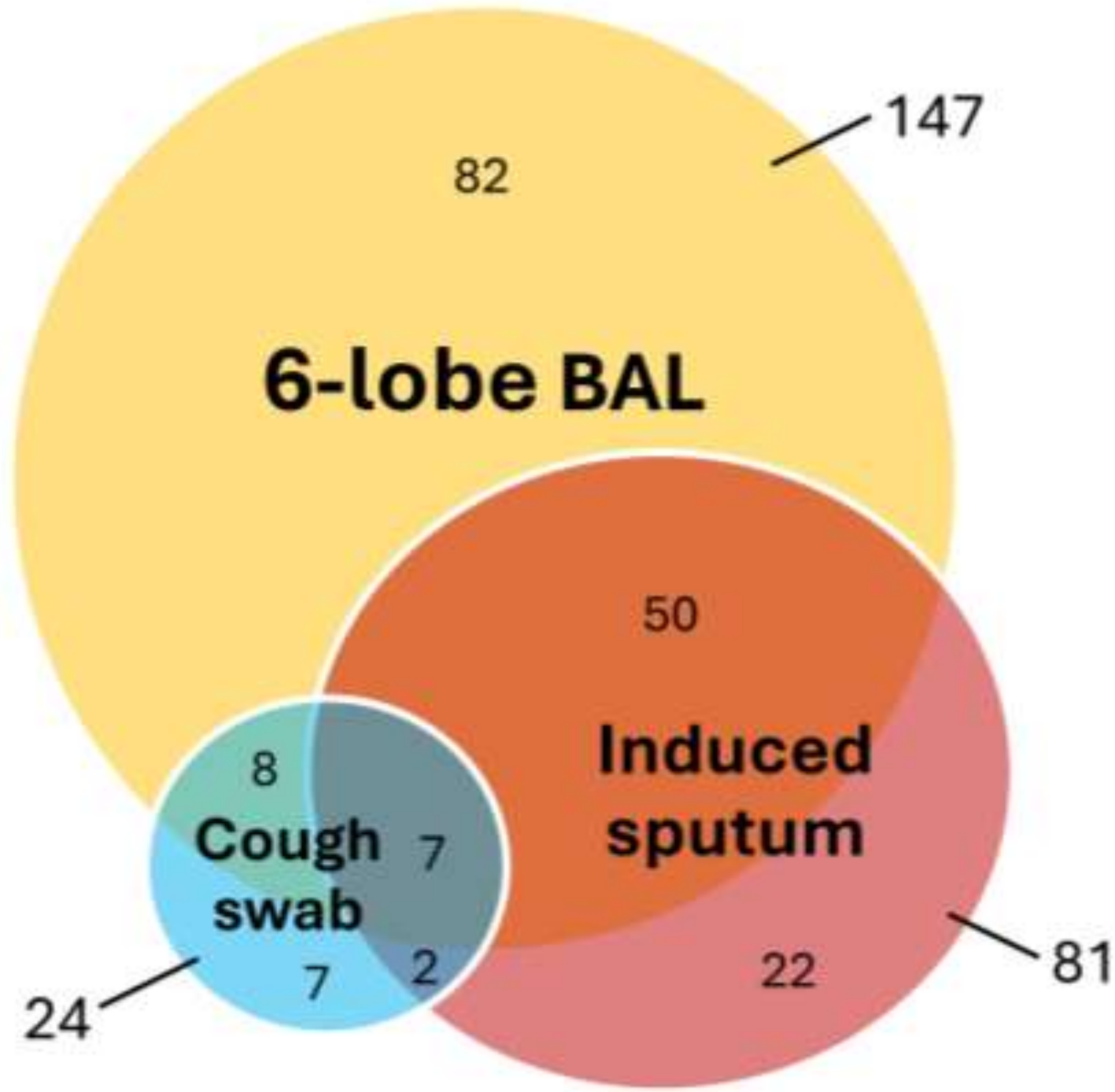

Supplement: Supplementary file 2 [file thorax-81-11-s002.pdf]
